# The Role of Biophysical Parameters in the Antilipopolysaccharide Activities of Antimicrobial Peptides from Marine Fish

**DOI:** 10.3390/md12031471

**Published:** 2014-03-13

**Authors:** Ramamourthy Gopal, Chang Ho Seo, Yoonkyung Park

**Affiliations:** 1 Research Center for Proteineous Materials, Chosun University, Gwangju 501-759, Korea; E-Mail: ramagopa@gmail.com; 2 Department of Bioinformatics, Kongju National University, Kongju 314-701, Korea; E-Mail: chseo@kongju.ac.kr; 3 Department of Biotechnology, Chosun University, Gwangju 501-759, Korea

**Keywords:** anti-lipopolysaccharide factor, LPS-binding domain, antimicrobial peptide, marine fish, innate immunity

## Abstract

Numerous antimicrobial peptides (AMPs) from marine fish have been identified, isolated and characterized. These peptides act as host defense molecules that exert antimicrobial effects by targeting the lipopolysaccharide (LPS) of Gram-negative bacteria. The LPS-AMP interactions are driven by the biophysical properties of AMPs. In this review, therefore, we will focus on the physiochemical properties of AMPs; that is, the contributions made by their sequences, net charge, hydrophobicity and amphipathicity to their mechanism of action. Moreover, the interactions between LPS and fish AMPs and the structure of fish AMPs with LPS bound will also be discussed. A better understanding of the biophysical properties will be useful in the design of AMPs effective against septic shock and multidrug-resistant bacterial strains, including those that commonly produce wound infections.

## 1. Introduction

Recent research has shown that certain marine peptides possess antimicrobial, antifungal, antiviral, anticoagulant and/or antifreeze properties; indeed, the number of antimicrobial peptides (AMPs) isolated from marine organisms and which are effective against pathogenic bacteria continues to grow. These AMPs are found in a wide range of phyla, including Crustacea, Cnidaria, Mollusca and Porifera, as well as in a number of fish species [1,2,3,4,5]. These include various marine fish species expressing anti-lipopolysaccharide factors (ALFs), so named because of their ability to specifically inhibit lipopolysaccharide (LPS)-mediated activation of the Limulus coagulation system [6,7]. 

ALFs were initially isolated from hemocytes of the horseshoe crabs (chelicerates) *Tachypleus tridentatus* and *Limulus polyphemus* [6,7] and were later identified in various other crustacean species, especially penaeid shrimp [2,8]. Crustacea and penaeidae species now known to express ALFs include *L setiferus* [9], *P. monodom* [10], *F. chinensis* [11], *M. japonicas* [12,13], *L. vannamei* [14,15], *F. paulensis*, *L. schmitti* [16] and *L. styrirostris* [17]. In addition, several ALF isoforms have been identified in *P. monodon* based on findings from the expressed sequence tag database [18]. Many of these ALFs exhibit similar antimicrobial and anti-LPS activities, and their amino acid sequences differ from those of other marine AMPs [19]. In marine fish, ALFs comprise an important part of the innate immune system that acts as the first line of defense against a broad spectrum of pathogens [20,21], especially Gram-negative bacteria, which express LPS. Furthermore, because LPS plays an important role in pathophysiological processes in humans [4,22,23,24,25,26,27,28,29,30], identifying and characterizing AMPs that are able to bind and/or neutralize LPS is a particularly attractive target in the effort to control Gram-negative bacterial disease.

LPS is the major component of the outer membrane of Gram-negative marine bacteria. Part of the function of LPS is to protect marine bacteria from harmful environmental factors. A major component of LPS is lipid A, also known as endotoxin. Lipid A molecules are the main pathogens responsible for septic shock in both fish and humans. When Gram-negative marine bacteria enter the body of a marine fish, Lipid A triggers the innate immune system via Toll-like receptors, which activates inflammatory signaling pathways that lead to septic shock. ALFs from marine fish are able to bind and neutralize lipid A, thereby preventing the inflammatory signaling [31,32]. These characteristics make ALFs a promising class of molecules for use in the development of drugs for the treatment of viral and bacterial diseases. In this review, we discuss the LPS binding domain of ALFs expressed in marine fish species as well as their structural amphipathicity (net cationicity and hydrophobicity) and other biological features, including their LPS binding/neutralization and antibacterial activity.

## 2. Anti-LPS Factors

### 2.1. StALF and PpALF2

The ALF isoform *Scylla tranquebarica* ALF (StALF) was isolated from hemocytes from the mud crab *Scylla tranquebarica*, while *Portunus pelagicus* ALF (PpALF2) was isolated from the blue swimmer crab *Portunus pelagicus*. Comparison of their sequences with those of previously characterized ALFs showed that both contain an LPS-binding domain [33]. The sequences of PpALF2 and StALF are rich in positively charged amino acid residues: both PpALF_2_ and StALF contain 12.4% lysine (Lys) and also contain 5.2% and 6.2% arginine (Arg), respectively. In addition, they contain substantial percentages of hydrophobic amino acids. Like other ALF molecules, PpALF_2_ and StALF exhibit a repeat pattern of cationic and hydrophobic amino acids within a disulfide loop, which suggests they share the same functional domain. Previous studies also reported that alternating hydrophilic and hydrophobic amino acids are characteristic of molecules with antimicrobial and LPS binding activities [34,35,36], and that hydrophobic amino acids are essential for the interaction between the peptide and LPS [37]. Moreover, the LPS binding domain of ALFs shows LPS neutralizing activity [12], and synthetic peptides corresponding to the crustacean ALF sequence show antimicrobial activity [38]. 

**Figure 1:**
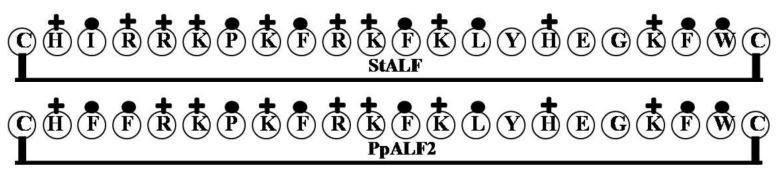
The surface of the hydrophobic and hydrophilic faces of the amphipathic PpALF2 and StALF. The PpALF2 and StALF have a repeat pattern of cationic and hydrophobic amino acids within a disulfide loop, which suggests they share the same functional domain. (+ = Basic residues; ●= Hydrophobic uncharged residues).

The 38 residues of the PpALF2 *N*-terminal region include 10 hydrophobic residues and 17 positively charged amino acids. Similarly, the 35 residues of the StALF *N*-terminal region contain 10 highly hydrophobic amino acids and 20 positively charged amino acids. The LPS binding domain is formed within a 22-residue disulfide loop (PpALF2L: CHFFRKPKFRKFKLYHEGKFWC, StALF: CHIRRKPKFRKFKLYHEGKFWC). The sequences of the loops are similar with the PpALF2 loop containing seven positively charged amino acids and a tryptophan (Trp) and the StALF loop containing nine positively charged amino acids and a Trp. The identical residues include C1, H2, R5, K6, P7, K8, F9, R10, K11, F12, K13, L14, Y15, H16, E17, G18, K19, F20, W21 and C22. However, residues 3 and 4 differ between the two loops: FF in PpALF *vs.* IR in StALF. Based on this difference in the LPS binding domain, the two molecules differ with respect to their molecular weights, net cationicity and percent hydrophobicity (Table 1). Nonetheless, the diagram in Figure 1 shows that the LPS binding domains of both PpALF_2_ and StALF are displaying cationicity, hydrophobicity and clustering of the cationic and hydrophobic motifs which are attributable to peptide’s amphipathic character. In addition, given their high similarity to other ALFs, the structural model of PpALF2 and StALF created using the SWISS-MODEL server consisted of two α-helices crowded against a four-strand β-sheet, like PpALF1 [39]. Importantly, an amphipathic loop is formed from the LPS binding domain situated between two β-strands [40]. This is an important factor conferring LPS-binding activity to ALFs [12].

**Table 1:** Cationic antimicrobial peptides within marine fish anti-lipopolysaccharide factors (ALFs).

Name of the Gene or Source	LPS Binding Domain	Net Charge	Hydrophilicity (%)	Hydrophobicity (%)	Molecular Weight
PpALF2	CHFFRKPKFRKFKLYHEGKFWC	8	45	36	2933.56
StALF	CHIRRKPKFRKFKLYHEGKFWC	9	50	32	2908.55
ALFSp	CHIRRKPKFRKFKLYHEGKFWC	9	50	32	2908.55
SpALF1	CHFRRRPKVRKFKLYHEGKFWC	9	50	32	2922.54
SpALF2	CHIRRRPKFRKFKLYHEGKFWC	9	50	32	2936.57
SsALF	CHIRRKPKFRKFKLYHEGKFWC	9	50	32	2908.55
PtALF	CYFRRRPKIRRFKLYHEGKFWC	9	50	32	2990.63
PtALF6	CNFRVMPRLRSWELYFRGDVWC	2	27	45	2834.37
ALFPm1	CRYSQRPSFYRWELYFNGRMWC	3	23	32	2949.43
ALFPm3	CKFTVKPYLKRFQVYYKGRMWC	6	27	36	2845.51
ALFPm6	CSFNVTPKFKRWQLYFRGRMWC	5	23	41	2854.44
MjALF1	CNFYVEPKFRNWQLRFKGRMWC	4	27	41	2909.47
MjALF2	CRYSQRPTFYRWELYFRGSMWC	3	23	32	2936.44
ALFFc	CKFTVKPYIKRFQLYYKGRMWC	6	27	36	2859.54
MrALF5	CSFQVKPRIKRWELYFRGTMWC	4	27	41	2835.44
MrALF6	CIYKRTGYFYKWELHYKAEVRC	4	36	27	2857.38
MrALF7	CTYNMRPFFKNWKLYYSASVIC	3	14	41	2735.27
ALFHa1	CRFSVKPTVRRFQLYFKGRMWC	6	27	41	2809.45
ALFHa2	CNFQVKPKIRRWQLYFVGSMWC	4	18	45	2790.38
ALFMolf	CQYSVTPRIKKLELWFKGRMWC	4	27	41	2773.4
EsALF	CNTRVMPTIKKFELYFRGRVWC	4	27	41	2748.36
LALF	CRKPTFRRLKWKIKFKFKC	9	47	37	2514.2
CRP19-1	CRKPTFRRLKWKIKGKFKC	9	47	32	2424.07
CRP19-2	CRKPTFRRLKWKYKGKFKC	9	47	26	2474.09
Pardaxin	GFFALIPKIISSPLFKTLLSAVGSALSSSGGQE	1	9	52	3323.9
Tachyplesin	KWCFRVCYRGICYRRCR	6	35	24	2268.82
Sushi 1	GFKLKGMARISCLPNGQWSNFPPKCIRECAMVSS	4	18	44	3757.53
Sushi 3	HAEHKVKIGVEQKYGQFPQGTEVTYTCSGNYFLM	2	24	29	3891.42
NRC-16	GWKKWLRKGAKHLGQAAIK	7	37	42	2176.61

### 2.2. ALFSp

**Figure 2:**
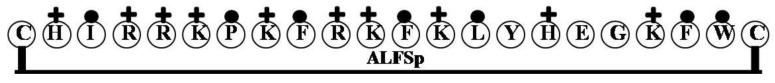
The surface of hydrophobic and hydrophilic faces of the amphipathic ALFSp. Designated ALFSp, it contains a 24-amino acids LPS binding site with a disulfide-bonded loop region the protein. (+ = Basic residues; ● = Hydrophobic uncharged residues).

An ALF from the mud crab *Scylla paramamosain* was identified and cloned using a cDNA library [38]. Designated ALF *Scylla paramamosai* (ALFSp), it contains a 24-amino acids LPS binding site (TCHIRRKPKFRKFKLYHEGKFWCP) with a disulfide-bonded loop region spanning residues 54–77 of the protein. Moreover, a synthetic 24-amino acid peptide consisting of the residues in the predicted LPS binding region exhibits bactericidal activity towards several species [38]. The ALFSp disulfide loop region contains a number of highly conserved basic amino acids (H56, R58, R59, K60, K62, R64, K65, K67, H70 and K73) and is situated in the middle of the protein sequence. In addition, there is a clustering of hydrophobic amino acids, including W75, in the *N*-terminal region of the protein [38]. This gives ALFSp an amphipathic character (Figure 2).

### 2.3. SpALF1 and SpALF2

**Figure 3:**
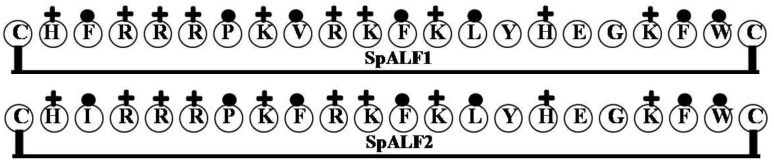
The surface of the hydrophobic and hydrophilic faces of the amphipathic SpALF1 and SpALF2. SpALF1 and SpALF2 from the mud crab *Scylla paramamosain* have been produced and the encoded proteins were expressed in *Pichia pastris*. (+ = Basic residues; ● = Hydrophobic uncharged residues).

The full-length cDNAs encoding SpALF1 (JQ069030) and SpALF2 (JQ06931) from the mud crab *Scylla paramamosain* have been produced [41] (Figure 3), and the encoded proteins were expressed in *Pichia pastris*. Comparative analysis of the recombinant proteins (rSP-ALF1 and rSp-ALF2) indicates the presence of a putative LPS binding loop, and synthetic peptide fragments of the LPS binding domain ((sSP-ALF1 (Acetylated-HTCHFRRRPKVRKFKLYHEGKFWCPG-aminated) and sSp-ALF2 (Acetylated-HTCHIRRRPKFRKFKLYHEGKFWCPG-aminated) as well as the recombinant proteins (rSP-ALF1 and rSp-ALF2), themselves, exhibit strong bactericidal activity against Gram-positive and Gram-negative bacteria [41]. 

### 2.4. SsALF

**Figure 4:**
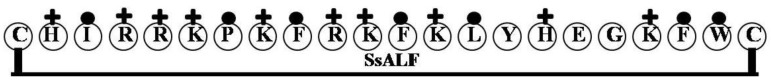
The surface of hydrophobic and hydrophilic faces of the amphipathic SsALF. SsALF has about 92% similarity with the ALFs and about 33%–53% amino acid sequence identify with other known ALFs. (+ = Basic residues; ● = Hydrophobic uncharged residues).

*Scylla serrata* ALF (SsALF) has been cloned, expressed with a Histag in *E. coli*, and characterized [42]. Sequence analysis showed that SsALF has up to 92% similarity with the ALFs from *Scylla paramamosain* and about 33%–53% amino acid sequence identify with other known ALFs. Expressed and purified recombinant SsALF exhibits both LPS neutralization and antimicrobial activity that involves bacterial membrane disruption [42]. Moreover, analysis of the putative LPS binding domain (CHIRRKPKFRKFKLYHEGKFWC) exhibited a strong positive charge of +9 with 32% hydrophobicity and an amphipathic structure (Figure 4). This structure is consistent with the idea that the highly cationic LPS binding domain binds to the highly anionic lipid A.

### 2.5. PtALF

**Figure 5:**
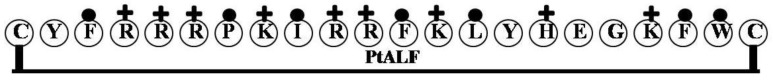
The surface of hydrophobic and hydrophilic faces of the amphipathic PtALF. PtALF has a clustering of hydrophobic and positively charged amino acid residues within a disulfide loop, giving the sequence the amphipathicity needed to bind and neutralize LPS. (+ = Basic residues; ● = Hydrophobic uncharged residues).

*Portunus trituberculatus* ALF (PtALF) from the swimming crab *Portunus trituberculatus* was first identified in a hemocyte cDNA library [43]. However, in response to infection with *Vibrio alginolyticus*, PtALF mRNA is ubiquitously expressed in muscle, stomach, heart, gill, hepatopancreas and hemocytes, suggesting it play an essential role in the animal’s innate immune response [43]. PtALF also contains a clustering of hydrophobic and positively charged amino acid residues within a disulfide loop (CYFRRRPKIRRFKLYHEGKFWC), giving the sequence the amphipathicity needed to bind and neutralize LPS [44] (Figure 5). In addition, the 22-residue of disulfide loop in which nine amino acids are positively charged residues and this peptide also contained a various hydrophobic residues Y56, F57, I63, F66 L68, Y69, F74 W75 [43]. Multiple alignment of the deduced amino acid sequence of PtALF showed 76%, 76%, 52% and 46% identity with the reported ALFs from *Scylla serrata*, *S. paramamosain*, *P. leniusculus* and *P. monodon*, respectively [43]*.* It is therefore likely that PtALF will share the anti-LPS and antibacterial activities of other ALFs [38,42].

### 2.6. PtALF6

**Figure 6:**
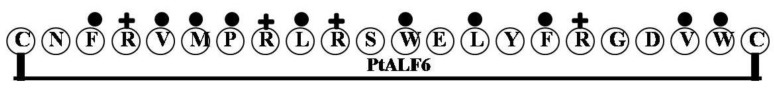
The surface of hydrophobic and hydrophilic faces of the amphipathic PtALF6. PtALF6 is composed of a signal peptide containing an LPS-binding domain within a disulfide loop. (+ = Basic residues; ● = Hydrophobic uncharged residues).

Another ALF from *Portunus trituberculatus*, *Portunus trituberculatus* ALF6 (PtALF6), was identified in an eyestalk cDNA library [45]. Like other ALFs, PtALF6 is composed of a signal peptide containing an LPS-binding domain within a disulfide loop. Following expression and purification of recombinant PtALF6, the protein showed antimicrobial activity, and the time-dependent expression of PtALF6 was accelerated after injection of *V. alginolyticus* into hemocytes, again indicating PtALF6 is significantly involved in the elimination of invasive pathogens [45]. Interestingly PtALF6 shares 84% sequence identity with ALFSp2, but there is only 10% amino acid identity between PtALF6 (27CNFRVMPRLRSWELYFRGDVWC48) and PtALF (CYFRRRPKIRRFKLYHEGKFWC) [45]. The LPS binding loop of PtALF6 contains four Arg residues, two Trp residues and a Lys residue, but unlike other LPS binding loops, it lacks a histidine (His). PtALF6 shows a net cationicity (+2) and 45% hydrophobicity with an amphipathic structure (Table 1 and Figure 6). The predicted tertiary structure of PtALF6 obtained using the SWISS MODEL showed a four-stranded anti-parallel β-sheet, an α-helix at the *N*-terminal and two α-helices at the *C*-terminal, including a hairpin loop formed by a disulfide bond (C27 and C48) between strands 2 and 3 of the anti-parallel β-sheet [45].

### 2.7. ALF-Pm3

Through nucleotide sequence analysis, two groups of ALFs have been derived from different genomic loci from the black tiger shrimp *Penaeus monodon*: Group A includes *Penaeus monodon* ALF1 (ALFPm1) and *Penaeus monodon* ALF2 (ALFPm2), while group B contains *Penaeus monodon* ALF3-5 (ALFPm3-5) [46]. Nearly all known mature ALF peptides contain about 100 amino acids, except ALFPm2, which contains only about 60 amino acids. Nonetheless, all ALFPms share the common features of being more hydrophobic at the *N*-terminal region than the *C*-terminal, and a conserved disulfide loop contains a positively charged cluster as the putative LPS-binding domain [47]. Of the five isoforms, the 98-residue ALFPm3, first identified in hemocytes [48], is the most abundantly expressed isoform of *P. monodon*. Earlier findings that ALFPm3 exerts its antibacterial effects by binding to LPS, which is why it is known as an ALF [49]; however, recombinant ALFPm3 exhibits bactericidal activity against both Gram-negative and Gram-positive bacteria [48]. Furthermore, recombinant ALFPm3 also shows bactericidal activity against *Vibrio harveyi* cells, acting through a membrane disruption mechanism [50], and is also able to eliminate white spot syndrome virus from *P. monodom* [51]. Tissue expression of both ALFPm2 and 3 was increased in response to *V. harveyi* infection, which is indicative of the important function of ALFs against bacterial invasion [46]. ALFPm3 also exerts an antiviral effect against human herpes virus (HSV-1) [52]. Interestingly, a synergistic effect was observed against bacterial strains exposed to a combination of ALFPm3 and shrimp lysozyme [53].

Biophysical techniques such as SPR indicate that rALFPm3 binds lipid A of LPS and OM-174, the water-soluble analogue of lipid A [54]. However, the structure of the complex formed by lipid A and the LPS binding site of rALFPm3 could not be determined using NMR due to their size. The secondary structure of ALFPm3 consists of three α-helices and a four-stranded β-sheet [54]. ALFPm3 and ALF-L have similar secondary structures, which include one disulfide bridge, although sequence identity between ALFPm3 and ALF-L is only 38.2% [47,54]. The disulfide bridge in ALFPm3 is essential for its structural stability; when the bridge is removed from ALFPm3 or ALF-L stability is lost [54]. To determine the lipid A-binding site in ALFPm3 and ALF-L, their structures were compared with that of FhuA. This analysis showed that ALFPm3, the ALF-L and FhuA share similar clusters of positively charged and hydrophobic residues gathered on their β-sheet surfaces. It is noteworthy that the entire β-sheet surface of FhuA is essential for lipid A-binding, which suggests that the lipid A-binding site (CKFTVKPYLKRFQVYYKGRMWC) of ALF-Pm3 and ALF-L are also β-sheet surfaces [54]. 

The ALFPm3 protein #35–51 contains part of a putative LPS-binding region (KPYLKRFQVYYKGRMWC) and shows some antimicrobial activity, but does not contain a disulfide bond. On the other hand, the synthetic LPS binding peptide clearly shows less antimicrobial activity towards Gram-negative bacteria than intact rALFPm3. It is noteworthy that reduction of the disulfide bond in the LPS-binding region of ALFPm3 protein #35–51 does not completely eliminate its antimicrobial activity; its effect on LPS binding activity is not yet known [48].

**Figure 7:**
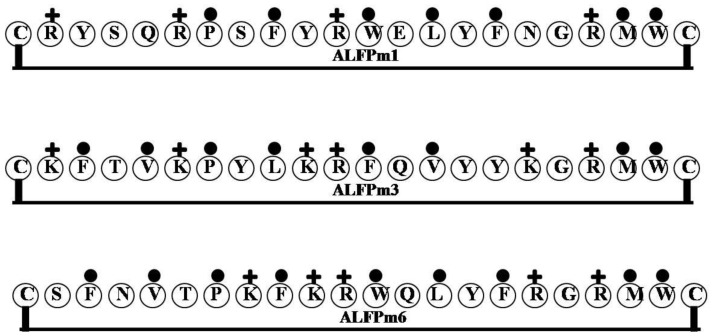
The surface of hydrophobic and hydrophilic faces of the amphipathic ALFs. ALFs: Group A includes ALF1 (ALFPm1) and ALF2 (ALFPm2), while group B contains ALF3-5 (ALFPm3-5). All ALFPms share the common features of being more hydrophobic at the *N*-terminal region than the *C*-terminal, and a conserved disulfide loop contains a positively charged cluster as the putative LPS-binding domain. ALFPm6 contain two Lys and two Arg. (+ = Basic residues; ● = Hydrophobic uncharged residues).

The amino acid composition of the putative LPS-binding domains of ALFPms differs somewhat. There are clear differences in the total numbers of positively charged amino acid residues (Arg and Lys) among the ALFPms. ALFPm1 (CRYSQRPSFYRWELYFNGRMWC) and ALFPm2 (CRYSQRPSFYRWELYFNGRMWC) contain four Arg, while ALFPm3 (CKFTVKPYLKRFQVYYKGRMWC), ALFPm4 (CKFTVKPYLKRFQVYYKGRMWC) and ALFPm6 (CSFNVTPKFKRWQLYFRGRMWC) contain two Lys and two Arg. Moreover, the LPS binding domain of ALFPm1, 2 and 6 contains two Trp residues, while those of ALFPm3 and 4 have only one Trp residue. Consequently, the degree of amphipathicity varies among ALFPms which is mainly based on the balance of the sizes of their cationicity and hydrophobicity (Figure 7).

### 2.8. MjALF1and MjALF2

cDNA encoding an ALF-like peptide (*Marsupenaeus japonicus* ALF (MjALF)) was isolated from the kuruma prawn *Marsupenaeus japonicus*. The deduced amino acid sequence of MjALF1 has 42% homology with the Japanese horseshoe crab *T. tridentatus* ALF, and Clustal W alignment showed 36.6% homology with L-ALF from the Atlantic horseshoe crab *L. polyphemus* [12]. MjALF1 mRNA is expressed in hemocytes, lymphoid organ, heart, intestine, muscle, stomach, hepatopancreas and gill. In one study, MjALF2 gene expression was found to be highest in heart and gill tissue [13]. By contrast, MjALF1 was more strongly expressed in hemocytes than other organs [12]. In addition, expression of MjALF1 mRNA in lymphoid organs was increased within 1.5–3 h after LPS administration, and then declined to baseline within 6 h. It thus appears that Mj-ALF1 is involved in the elimination of pathogens *in vivo* [12].

**Figure 8:**
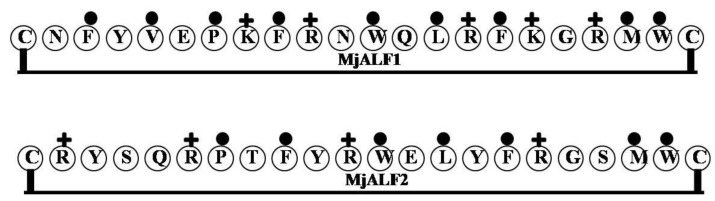
The surface of hydrophobic and hydrophilic faces of the amphipathic MjALF 1 and 2. MjALF1 and MjALF2 gene expression was found to be the highest in different sections. By contrast, the LPS binding sites of MjALF1 and MjALF2 share identical amino acids at C1, P7, F9, W12, L14, F16, G18, M20, W21 and C22. (+ = Basic residues; ● = Hydrophobic uncharged residues).

The LPS binding sites of MjALF1 (CNFYVEPKFRNWQLRFKGRMWC) and MjALF2 (CRYSQRPTFYRWELYFRGSMWC) share identical amino acids at C1, P7, F9, W12, L14, F16, G18, M20, W21 and C22. In addition, synthetic peptides containing amino acids C30 to C51, corresponding to the LPS-binding domain*,* efficiently neutralize LPS and inhibit NO production in RAW264.7 cells [12]. This suggests an amphipathic β-hairpin loop with an alternative pattern of hydrophilic (mainly basic amino acids K and R) and hydrophobic residues between C30 and C51 are able to bind LPS in a manner similar to other ALFs (Figure 8).

### 2.9. ALFFc

**Figure 9:**
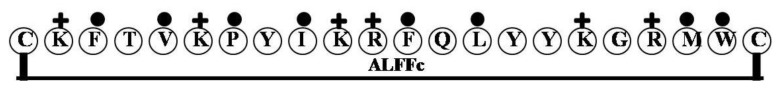
The surface of hydrophobic and hydrophilic faces of the amphipathic ALFFc. ALFFc showed the amino acid sequence of ALFFc shares 56% homology with the sequences of ALFs from *Tachypleus tridentatus* and *L. polyhemus*. ALFFc contains a disulfide loop that binds LPS and neutralizes its septic effects.

The full-length cDNA encoding ALF *Fenneropenaeus chinensis* (ALFFc) was cloned from hemocytes from the Chinese fleshy prawn *Fenneropenaeus chinensis*. Multiple alignments showed the amino acid sequence of ALFFc shares 56% homology with the sequences of ALFs from *Tachypleus tridentatus* and *L. polyhemus*. ALFFc contains a disulfide loop (CKFTVKPYIKRFQLYYKGRMWC) that binds LPS and neutralizes its septic effects [12,55,56,57,58,59]. ALFFc transcripts are mainly detected in hemocytes, gill and intestine, and ALFFc expression is significantly enhanced in response to *V. anguillarum* infection [11] (Figure 9).

### 2.10. MrALF5, MrALF6 and MrALF7

**Figure 10:**
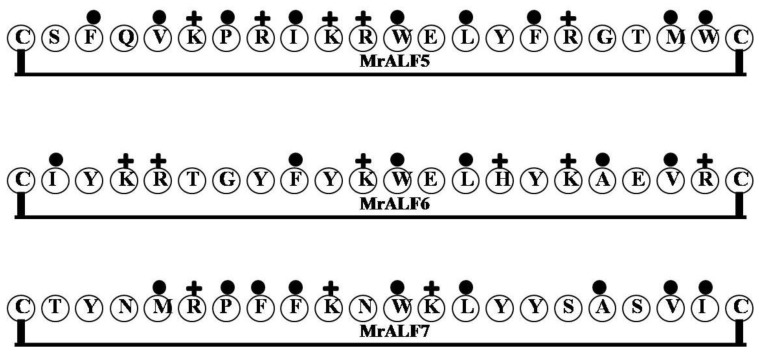
The surface of hydrophobic and hydrophilic faces of the amphipathic MrALF. Multiple isoforms of MrALF have MrALF5, MrALF6 and MrALF7. The isoforms of MrALF, MrALF5-7 contain a LPS-binding domain with two conserved Cys residues and eight conserved hydrophobic amino acids; but, their amino acid compositions, and degrees of amphipathicity all differ from one another.

Multiple isoforms of *Macrobrachium rosenbergii* ALF (MrALF) have been identified in the giant freshwater prawn *Macrobrachium rosenbergii*. Of them, MrALF5 (CSFQVKPRIKRWELYFRGTMWC), MrALF6 CIYKRTGYFYKWELHYKAEVRC and MrALF7 (CTYNMRPFFKNWKLYYSASVIC) have been characterized [60]. MrALF5 is expressed mainly in the hepatopancreas, gills and heart, whereas MrALF6 was mainly distributed in the intestine and hepatopancreas, and MrALF7 is strongly expressed in the hepatopancreas. MrALF6 and MrALF7 were downregulated by *E. coli* challenge. By contrast, MrALF5-7 gene expression is upregulated by *Vibrio* or white spot syndrome virus challenge, suggesting MrALF5-7 may participate in the immune response to the bacteria and virus. Importantly, MrALF5-7 contain a LPS-binding domain with two conserved Cys residues and eight conserved hydrophobic amino acids; however, their amino acid compositions, balance of net charge and hydrophobicity and degree of amphipathicity all differ from one another (Table 1 and Figure 10).

### 2.11. ALFHa1 and ALFHa2

**Figure 11:**
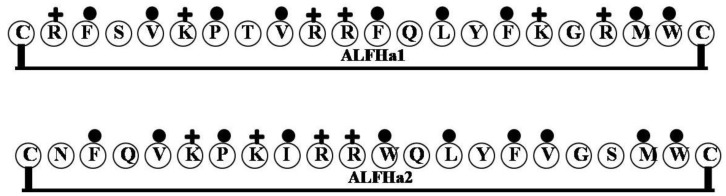
The surface of hydrophobic and hydrophilic faces of the amphipathic ALFHa1 and ALFHa2. The LPS binding domain of ALFHa1 contains four Arg and two Lys, while that of ALFHa2 has two Arg and two Lys. Consequently, the LPS binding domain of ALFHa1 is considerably more cationic than that of ALFHa2.

ALF *Homarus americarius*1 (ALFHa1) and ALF *Homarus americarius*2 (ALFHa2) were identified from the American lobster *Homarus americarius* [61]. The sequences of both contain an *N*-terminal signal peptide and Cys residues participating in a disulfide bridge. The gene expression of ALFHa1 was increased by *Vibrio fluvialis* in the gill, hemocytes and hepatopancreas, whereas expression of ALFHa2 was not significantly affected by *Vibrio* in any of the three tissues tested [61]. The LPS binding domain of ALFHa1 (CRFSVKPTVRRFQLYFKGRMWC) contains four Arg and two Lys, while that of ALFHa2 (CNFQVKPKIRRWQLYFVGSMWC) has two Arg and two Lys (Figure 11). Consequently, the LPS binding domain of ALFHa1 is considerably more cationic than that of ALFHa2. The LPS binding/neutralization activities of the two LPS binding domains have not yet been determined using synthetic peptides under *in vitro* assay conditions. 

### 2.12. ALFMolf

**Figure 12:**
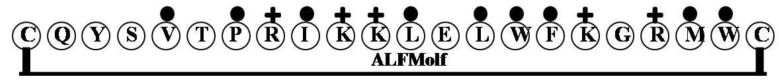
The surface of hydrophobic and hydrophilic faces of the amphipathic ALFMolf. The LPS binding domain of ALFMolf contained two Arg and three Lys including two Trp.

The sequence of an ALF *Macrobrachium olfersi* (ALFMolf*)* was identified in hemocytes from the freshwater prawn *Macrobrachium olfersi* [16]. The amino acid sequence encoded by the ALFMolf cDNA showed homology to other ALFs from limulids and crustaceans. The LPS binding domain of ALFMolf (CQYSVTPRIKKLELWFKGRMWC) contained two Arg and three Lys as well as a number of hydrophobic amino acids, including two Trp [16] (Figure 12). Early studies indicated that LPS binding motifs in host defense proteins were formed by amphipathic sequences rich in cationic and hydrophobic residues [47,55]. The KGRMWC sequence in the LPS binding domain of ALFMolf showed homology to those from ALFFc, ALFPm3 and MjALF. 

### 2.13. EsALF

**Figure 13:**
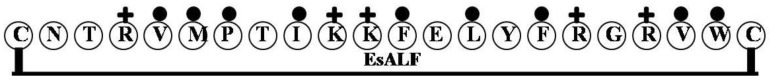
The surface of hydrophobic and hydrophilic faces of the amphipathic EsALF. The LPS binding domain in EsALF contains three Arg and two Lys.

The cDNA encoding *Eriocheir sinensis* ALF (EsALF) was identified in the Chinese mitten crab *Eriocheir sinensis* [62]. The mature EsALF peptide was expressed in *E. coli*, and the recombinant EsALF exhibited bactericidal activity towards a spectrum of bacterial strains that was similar to recombinant ALFs from *P. monodon* [47] and *T. tridentatus* [63]. EsALF mRNA was mainly expressed in hemocytes, heart and gonad, and less strongly in gill, eyestalk and muscle, and expression of EsALF mRNA was increased in response to *Vibrio anguillarum* infection. The sequence of the LPS binding domain in EsALF (CNTRVMPTIKKFELYFRGRVWC) contains several cationic (three Arg and two Lys) and hydrophobic residues for LPS binding activity (Figure 13).

### 2.14. LALF

**Figure 14:**
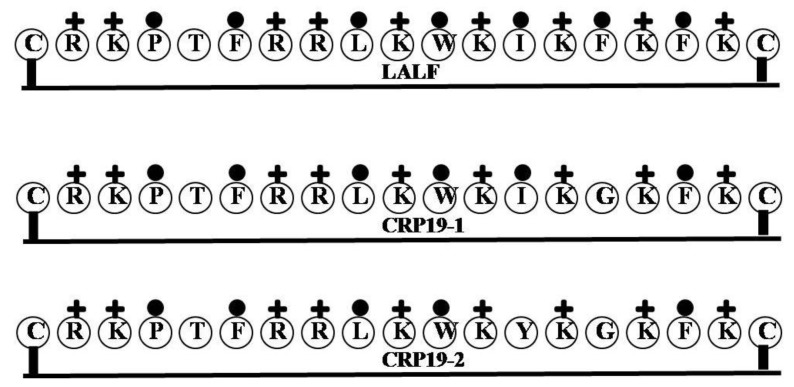
The surface of hydrophobic and hydrophilic faces of the amphipathic LALF, CRP19-1 and CRP19-2. CLP19 is a synthetic peptide derived from Limulus ALF, which neutralizes LPS toxicity, inhibits LPS-induced TNF-α production in PBMCs and protects mice from LPS-induced shock *in vivo*. Furthermore, two analogs of CRP19, CRP19-1 and CRP19-2, were synthesized to assess the effect of altering the hydrophobicity of the parent molecule.

The Limulus Anti-LPS factor was identified in hemocytes from the marine chelicerates *Tachypleus tridentatus* and *Limulus polyphemus*. The sequence between amino acids 31 and 52 (CRKPTFRRLKWKIKFKFKC) comprises the LBS binding domain [64]. CLP19 is a synthetic peptide derived from Limulus ALF, which neutralizes LPS toxicity, inhibits LPS-induced TNF-α production in peripheral blood mononuclear cells (PBMCs) and protects mice from LPS-induced shock *in vivo* [65]. It has also been reported that CLP19 is able to reduce secretion of TNF-α from PBMCs stimulated with LPS in the presence of human serum [65], and to block LPS induced phosphorylation of the MAPK signaling proteins p38, ERK1/2, ABD and JNK1/2 in PBMCs [65]. In addition, two analogs of CRP19, CRP19-1 (CRKPTFRRLKWKIKGKFKC) and CRP19-2 (CRKPTFRRLKWKYKGKFKC), were synthesized to assess the effect of altering the hydrophobicity of the parent molecule [66]. The order of hydrophobicity was CRP-19 > CRP19-1 > CRP19-2. When the ability of the three molecules to neutralize LPS, inhibit TNF-α release from LPS-simulated RAW 264.7 cells, and protect mice from endotoxemia were compared, CRP19-2 showed less activity than CRP19-1, which in turn showed less activity than CRP-19. Clearly then, reducing hydrophobicity reduces the peptides ability to inhibit LPS [66], which is consistent with the idea that the hydrophobicity of the factor is important for binding LPS [37,55] (Figure 14). 

### 2.15. Pardaxin

**Figure 15:**
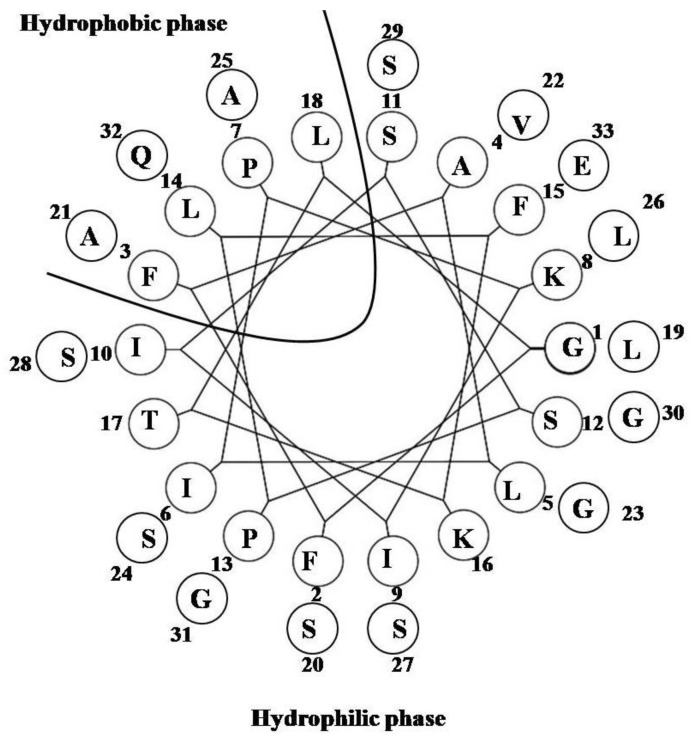
The surface of hydrophobic and hydrophilic faces of the amphipathic pardaxin. Pardaxin is an amphipathic polypeptide composed of 33 amino acid residues showing a broad spectrum of antibacterial activities were also found in the mucous glands of sole fish.

Pardaxin is an amphipathic polypeptide composed of 33 amino acid residues (GFFALIPKIISSPLFKTLLSAVGSALSSSGGQE) which was identified in the pacific peacock sole fish *Pardachirus pavoninus* [67] and the red sea moses sole fish *Pardachirus marmoratus* [68] (Figure 15). Later, pardaxins (Pa1, Pa2, Pa3 and Pa4) showing a broad spectrum of antibacterial activities were also found in the mucous glands of sole fish [69]. The three dimensional structure of Pa4 with LPS has been resolved using NMR [69], which showed the horseshoe structure of Pa4 in LPS micelles. The Lys residues present at positions 8 and 16 of Pa4 interact with the lipid A moiety of LPS via a salt bridge or hydrogen bond. Lys8 is located in a *N*-terminal short helix (residues Leu5-Ser12) and Lys16 is in a *C*-terminal longer helix (residues Lys16-Ser28). Residues Pro13, Leu14 and Phe 15 form a short loop between the short helix and the longer helix. Other residues, Phe2-Ala4 and Ser29-Glu33, fall into extended conformations in the LPS micelle [69]. This characteristic helix loop helix structure formed in the LPS micelle is essential for interaction with LPS. Moreover, deleting the charged residues Lys8/Lys16 or the aromatic residue Phe15 from Pa4 reduces its interaction with the bacterial membrane and its antibacterial activities [70,71]. Thus Lys8/Lys16 and phe15 with the helix loop helix configuration are important for membrane disruption and the antibacterial activity of Pa4. Another recent study showed that the pardaxin GE33 protects against methicillin-resistant *S. aureus* (MRSA) infection in mice with skin injuries. GE33 reduced the amounts of two pro-inflammatory mediators, TNF-α and IL-6, released during wound healing, thereby promoting the wound healing process [72]. 

### 2.16. Tachyplesin

**Figure 16:**
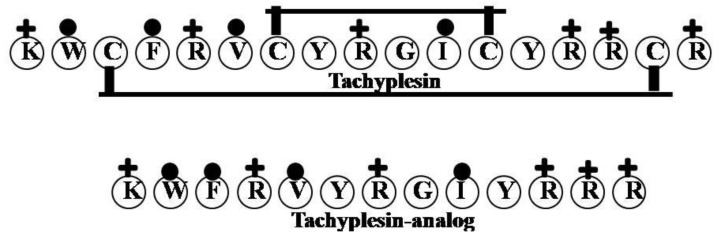
The surface of hydrophobic and hydrophilic faces of the amphipathic tachyplesin. The NMR structure showed that a tachyplesin-analog was stabilized in LPS micelles through the interaction between the aromatic ring of W2 and the side chain of the nonpolar amino acid V5 and the cationic side chain of R11. Furthermore, the tachyplesin-analog displays an extended positively charged surface patch composed of residues R4, R7, R12 and R13.

Anti-LPS tachyplesin was isolated from the hemocytes of the horseshoe crab [73]. Using NMR, the structure of tachyplesin-1 (KWCFRVCYRGICYRRCR-NH_2_) with LPS was shown to contain a disulfide-stabilized β-hairpin. Another study of the NMR structure showed that a tachyplesin-analog (KWFRVYRGIYRRR-NH2) was stabilized in LPS micelles through the interaction between the aromatic ring of W2 and the side chain of the nonpolar amino acid V5 and the cationic side chain of R11 [74]. Apart from the structure of the β-hairpin, the tachyplesin-analog displays an extended positively charged surface patch composed of residues R4, R7, R12 and R13 (Figure 16). Salt bridges, electrostatic interactions and hydrogen bonds would be expected between these residues and the anionic phosphate groups of LPS, which would stabilize the β-hairpin structure of the tachyplesin-analog [74], while the hydrophobic residues of tachyplesin-1 would interact with the acyl chains of LPS [75]. It is noteworthy that despite the absence of a disulfide bond, the tachyplesin-analog retained the ability to bind LPS and disrupt the bacterial cell membrane [74].

### 2.17. Sushi Peptides

**Figure 17:**
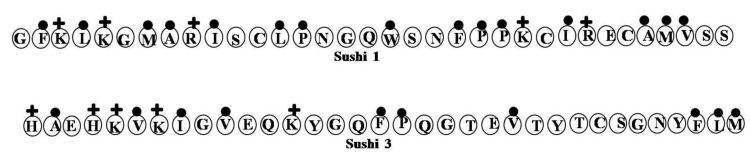
The surface of hydrophobic and hydrophilic faces of the amphipathic Sushi 1 (S1) and Sushi 3 (S3). S1 and S3 peptides were interact significantly with LPS. Sequence analysis indicates that S1 and S3 have larger numbers of Lys and Arg at their *N*-terminal and more hydrophobic residues at their *C*-terminal.

Sushi 1 (S1, GFKLKGMARISCLPNGQWSNFPPKCIRECAMVSS) and Sushi 3 (S3, HAEHKVKIGVEQKYGQFPQGTEVTYTCSGNYFLM) were derived from the LPS-binding domain of an LPS-sensitive serine protease, Factor C, from the horseshoe crab [76,77] (Figure 17). Both peptides interact significantly with LPS. Sequence analysis indicates that S1 and S3 have larger numbers of Lys and Arg at their *N*-terminal and more hydrophobic residues at their *C*-terminal. The positively charged Lys and Arg residues contribute to electrostatic interactions with the diphosphoryl head groups of the LPS, while the hydrophobic residues most likely interact with the hydrophobic acyl chains of the LPS though hydrophobic interactions. Another study also explained that the extra two Lys residues at the *N*-terminus of the peptide likely increase the peptide’s LPS-neutralizing activity [77]. The disulfide bond is highly important in the S3 peptide, as its reduction makes S3 incapable of LPS binding. In addition, fluorescence COSY assays showed that the disulfide bond in the dimeric S3 peptide has detergent-like properties and is involved in the disruption of LPS micelles [24]. 

**Figure 18:**
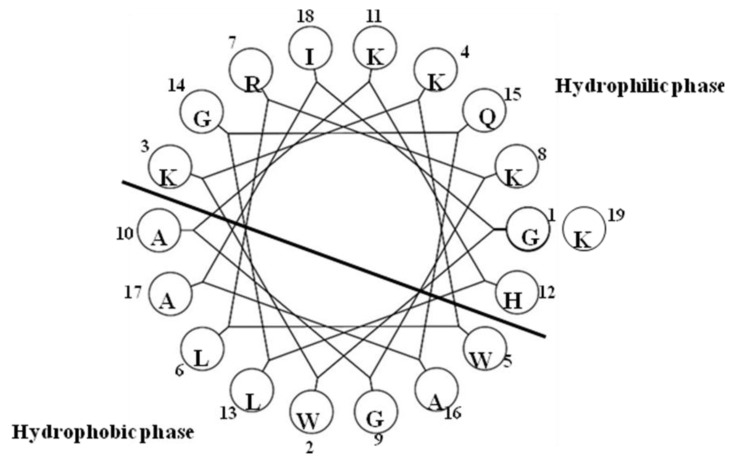
The surface of hydrophobic and hydrophilic faces of the amphipathic NRC-16. NRC-16 contains four polar uncharged amino acids, seven polar charged residues and eight hydrophobic residues. These amino acids have enough potential to provide the amphipathicity necessary for LPS binding.

### 2.18. Pleurocidin-Like Cationic AMP

The NRC-16 peptide (GWKKWLRKGAKHLGQAAIK-NH_2_) [78] is a truncated form of NRC-17 (GWKKWLRKGAKHLGQAAIKGLAS), which was identified in the witch flounder *Glyptocephalus cynoglossus* [79]. NRC-16 contains four polar uncharged amino acids, seven polar charged residues (five Lys, one Arg and one His) and eight hydrophobic residues (two Trp, three Ala, two Leu and one Ile) (Figure 18). These amino acids have enough potential to provide the amphipathicity necessary for LPS binding [78]. This peptide thus binds LPS and exhibits a helical structure in the LPS micelles [80], which is consistent with other AMPs that adopt helical structures in LPS lipids [69,81,82,83,84,85]. 

## 3. Functions of Cationic ALFs

The cationic ALFs with various potentials to form amphipathic α-helices, β-sheet and loop are rich in cationic and hydrophobic side chains. Particularly, the LPS binding domain of ALFs mostly adopt β-sheet with amphipathic loop. The other ALFs (NRC-16 and pardaxin) adopted amphipathic α-helices with LPS. This clearly explains that the cationic and hydrophobic residues, hydrophobicity, amphipathic property and secondary structures are needed for the LPS binding domain of the ALFs. Moreover, some of the ALFs discussed in this review bind LPS and exhibit both anti-LPS and antimicrobial activity, but are not sufficient to neutralize LPS-induced macrophage activation. Others were capable of binding to pathogens and killing bacteria, exhibiting both LPS sequestering and neutralization, though their mechanism of action is not yet known. It is believed that AMPs (e.g., cathelicidins and HPA3P2) are able to tightly bind LPS and break its aggregates [86,87], which blocks the binding of LPS to its carrier, LPS binding protein, or to its receptor, CD14, thereby suppressing the production of cytokines by RAW264.7 cells or bone marrow-derived primary macrophages. ALFs have also been shown to suppress LPS-induced pro-inflammatory responses *in vivo*, and to protect against sepsis in animal models [87]. Atlantic salmon cathelicidin (asCATH2), for example, exhibits antimicrobial action at the site of pathogen invasion (e.g., epithelial surfaces) without inducing hemolysis and stimulates expression of the chemokine IL-8, which suggests cathelicidins may play an immunomodulatory role in fish [88]. 

LPS also contributes directly and indirectly to bacterial biofilm formation [89,90] and is involved in mediating the cohesion and stabilization of bacterial biofilms. Indeed, the absence of LPS alters biofilm formation and reduces adhesion [91], as compared to biofilms from LPS-producing strains [92,93,94]. For instance, LPS is essential for colonization of *Arabidopsis thaliana* hydathodes by *Xanthomonas campestris*, a Gram-negative bacterium that is a common plant pathogen [95].

It is clear that ALFs are essential components of innate immunity in fish, contributing to the first line of defense against a wide range of pathogens. AMPs and ALFs are capable of binding and neutralizing LPS and promoting angiogenesis and wound healing [96,97]. It has been reported that rationally designed new synthetic anti-LPS peptides (SALPs) based on the Limulus anti-LPS factor, showed LPS binding/neutralization and blockade of its immunopathological consequences *in vitro* and *in vivo*. In addition, these peptides exhibit very low cytotoxicity under physiological conditions, making SALPs promising candidates for application as therapeutic agents for the prevention and treatment of septic shock [98].

## 4. Conclusions

Because of their antimicrobial, antibiofilm, anti-inflammatory, and pro-angiogenesis and wound healing activities, there is growing interest in the therapeutic potential of ALFs. It is anticipated that the design of new AMPs based on the LPS binding domain of ALFs will enable development of novel synthetic peptides with antimicrobial, anti-inflammatory and anti-biofilm activities in a wound environment.
